# Working Conditions Influencing Junior School Principalship as a Satisfying Profession: A Cross-Country Comparative Study

**DOI:** 10.3389/fpsyg.2022.834349

**Published:** 2022-03-25

**Authors:** Bo Ning, Hongqiang Liu, Yiming Cui

**Affiliations:** ^1^Research Institute for International and Comparative Education, Shanghai Normal University, Shanghai, China; ^2^School of Foreign Languages, Henan University of Technology, Zhengzhou, China

**Keywords:** school principal, professional satisfaction, workplace environment satisfaction, reward satisfaction, workload stress

## Abstract

Although prior studies have extensively investigated the effect of working conditions upon professional satisfaction, the cross-national variance in the effect remains largely understudied due to technical or financial restrictions. The Teaching and Learning International Survey (TALIS) provides an opportunity to investigate the cross-country differences in the impact of working conditions upon principals’ professional satisfaction. The current study attempts to investigate the overall and specific effects of principals’ workplace environment satisfaction, rewards satisfaction, and workload stress on their professional satisfaction in 47 countries included in the TALIS 2018. The results indicate that workplace environment, rather than the typically regarded effort-reward issues, is the most powerful contributor to individual principals’ professional satisfaction across 47 countries, while, in countries with low overall professional satisfaction, rewards satisfaction and workload stress are more powerful predictors for principals’ professional satisfaction on the country level. This study may inform policymakers in school principal stimulation and retention that effective practice to stimulate and/or retain school principals may vary across countries and research findings derived from data on the individual level may not be applicable to practice on the country level.

## Introduction

A main concern for policymakers and researchers in human resources management is to sustain and satisfy qualified employees with their job. Within the field of job satisfaction research, however, the discussion about what job satisfaction remains contentious (see Aziri, 2011 for an overview). Operationally, job satisfaction could be considered as “a function of the perceived relationship between what one wants from one’s job and what one perceives it is offering” (Locke, 1969, p.309). Meanwhile, research evidence has shown the relevance of distinguishing the profession from the working conditions in job satisfaction. For instance, teachers tend to express satisfaction with elements directly related to the teaching profession that can fulfill their personal values and goals, while showing dissatisfaction with working conditions, such as salary and workload (Mostafa and Pál, 2018; Viac and Fraser, 2019). In this study, professional satisfaction reflects principals’ satisfaction with the content and construct of the work itself, that is, the experience of working as a professional, while workplace environment satisfaction, rewards satisfaction, and workload stress reflect principals’ satisfaction with their working conditions.

Good working conditions in general attract competitive workers and contribute to their career success and occupational well-being (Eurofound and International Labour Organization, 2019). According to the International Labour Organization (2022), typical working conditions include work time, rewards, physical conditions, and mental demands in the workplace, which are the core of paid work and employment relationships. Similarly, improving working conditions is an important goal of the European Union (2012). The European Foundation for the Improvement of Living and Working Conditions, 2012) defined working conditions as the workplace environment and aspects of an employee’s terms and conditions of employment, particularly, the organization of work and work activities; training, skills and employability; health, safety and wellbeing; working time and work-life balance; and rewards.

Prior studies on job satisfaction in education domain have mainly focused on the relationship between teachers’ professional satisfaction and turnover, commitment, and even student outcomes (Ronfeldt et al., 2013; Dupriez et al., 2016; Dicke et al., 2018a), but overlooked school principals’ professional satisfaction, particularly its relationship with their working conditions (Darmody and Smyth, 2016). Still, researchers in job satisfaction can hardly reach a consensus on the properties of working conditions leading to employees’ professional satisfaction. The current study aims to contribute to the literature via exploring the relationship between principals’ professional satisfaction and working conditions measured by workplace environment, rewards, and workload stress, in 47 countries participating in the 2018 Teaching and Learning International Survey (TALIS 2018).

The rationale behind this study is that principals’ work is quite heavy and complex and quires their independent judgment and initiatives in many countries (Organisation for Economic Co-operation and Development, 2019b). Their satisfaction with working conditions stimulate their devotion to the profession (Eurofound and International Labour Organization, 2019). To benefit and sustain principals’ professional satisfaction, policymakers and the society need to know the readiness and efficiency of diverse working conditions provided for school principals in domestic countries. More specifically, if these working conditions compete with one another due to the limited public budget, policymakers need to know the priority.

### Principals’ Professional Satisfaction in the Multidimensional Structure of Job Satisfaction

A cross-country comparative study contributes to deeper understanding of principals’ job satisfaction. Although the strong call for principal professionalization, particularly for the benefits of school effectiveness and school improvement, in many countries (Alfirevic et al., 2018), whether and to what extent principalship is a well-defined profession is still contentious (Karataş, 2019). This disagreement is in part attributable to the principals’ different distribution of time at work across countries. For instance, the results from TALIS 2013 survey indicated that junior secondary school principals differed largely in their time allocation to administrative, instructional, and interactive tasks across countries, where principals’ main roles and responsibilities might be formed differently by socio-cultural norms and school administration systems (Ning, 2021). Partly due to this reason, a school principal might be recognized as a schoolmaster, school manager, or headteacher in different countries.

In studies on principals’ job satisfaction, researchers usually conceptualize job satisfaction as a multidimensional construct comprising the global idea of the profession, indicating his or her work experience as a professional, as well as specific working condition elements, such as, the workplace environment, rewards, and workload stress (see Organisation for Economic Co-operation and Development, 2019a), while all of them can be examined as interconnected elements (Darmody and Smyth, 2016). Typically, there are two schools of thought in the prime determinant of job satisfaction (Judge et al., 2017). According to the Tavistock Institute staff, the profession itself is the prime determinant of job satisfaction; this perspective is known as the work content and job design model. Similarly, Hackman and Oldham (1976) formulated the job characteristics model, which summarized satisfied jobs as five core characteristics (variety, autonomy, feedback, significance, and identity) and three critical psychological functions (experiencing meaning, feeling responsible for outcomes, and understanding the results of their efforts).

Conversely, according to the Manchester Business School, the main determinants of job satisfaction are professional workload and job-related rewards; this is the effort-reward bargain model. Furthermore, the diverse working conditions are interdependent and mutually conditional to one another in job satisfaction. For instance, the lack of reciprocity between high effort at work and low benefits from work produces job stress (Theorell, 2017) afflicting workers’ health and well-being (Montano and Peter, 2021). Another study indicated that workers’ perception of workplace environment and workloads related to their decision to attend to scheduled work (Montano, 2020). Also, the psychosocial characteristics of workplace environment related to workload stress (Bowling et al., 2015).

Generally, principals’ professional satisfaction, an assessment of the favorability of their work, is important for their well-being and job retention, both of which are crucial in school effectiveness and improvement (Dicke et al., 2020). Some researchers have argued that the most important motivation for school principals to join and remain employed in this field is the sense of fulfillment they derive from serving the public (e.g., influencing children’s development and contributing to society) (Grissom and Brendan, 2018). Compared with the work itself, however, working conditions are more amenable to influences from policy. Policymakers used to regulate workplace environment, rewards, and workload stress that are influential to principals’ professional satisfaction in their respective countries, taking into consideration their stable duties and professional requirements (Yan, 2020). Additionally, it should be noted that working conditions (how the employees are treated by their employers) differ from the school context, which includes community economy and culture, student and teacher population and composition, school resources and governance, and general educational expenditure (Yan, 2020).

Principals’ professional satisfactions differ across countries. The 2018 Teaching and Learning International Survey (TALIS 2018) results indicated high professional satisfaction among principals in countries like Austria, Argentina (in Buenos Aires, specifically), Chile, Colombia, Denmark, Estonia, Israel, Korea, Mexico, Netherlands, Singapore, Slovenia, Spain, the United Arab Emirates, the United States, and Viet Nam. Conversely, in Bulgaria and Belgium (specifically among individuals of the French community), only 40% of principals stated that the advantages of the profession outweighed the disadvantages, compared with the average of countries in the Organisation for Economic Cooperation and Development (OECD), which was 81%. Likewise, more than 20% of principals reported regretting their decision to become principals in Bulgaria, Canada (in Alberta, specifically), Saudi Arabia, and Turkey, compared to the OECD average of 7%. In addition, more than 30% of principals in Bulgaria, Lithuania, Malta, Saudi Arabia, South Africa, and Turkey reported that they wondered whether it would have been better to choose another profession, compared to the OECD average of 20%. The number of principals who agreed with this statement was particularly high in Lithuania (77%) and Saudi Arabia (50%). It is worthwhile to explore the working conditions related to principals’ professional satisfaction from a cross-country comparative perspective, which provides policy clues from both domestic and international experiences.

### Working Conditions Related to Principals’ Professional Satisfaction

The influence of rewards satisfaction, particularly salary satisfaction, on school principals’ professional satisfaction and retention has been researched extensively over the last decades (see Tran, 2017; Yan, 2020). Schools and education systems need to offer attractive rewards, in terms of salary and contracts, to their staff (Organisation for Economic Co-operation and Development, 2019b). In countries included in TALIS 2018, around 47% of school principals reported being satisfied with their salary, with those in private schools being more satisfied (65%) than their counterparts in public schools (42%). Comparatively, school principals were more satisfied with their work contracts (66%) than with their salaries (47%). In considering what constitutes an attractive salary, various elements should be taken into account: the regular items concerning basic and performance salary based on personal qualifications and professional duties, the supplemental items concerning annual increases related to work experience and contributions, the purchasing power of principals’ salary, and the relative level of principals’ salary compared to other careers with similar education and professional status (Organisation for Economic Co-operation and Development, 2019b).

A recent study in the United States indicated that an improvement in principals’ salary and job benefits (such as tenure and job contracts) significantly decreased the probability of turnover in K-12 public schools, even when controlling for principals’ background and school context (Yan, 2020). However, when controlling for other working conditions, such as workplace environment and workload, the effect of salary on turnover was reduced and became non-significant (Yan, 2020). In line with this study, Maforah and Schulze (2012) found that more than two-thirds of principals in disadvantaged secondary schools in South Africa were dissatisfied with their salary, which was the most important predictor for their dissatisfaction with the profession. Many principals in these disadvantaged schools argued that the salary was not up to the effort they invested and that the salary was insufficient to cover their needs (Maforah and Schulze, 2012). An interesting finding in this study is that only a few principals considered salary as a reason for staying at their current jobs; thereafter, receiving a poor salary may not be a reason strong enough for them to leave the profession (Maforah and Schulze, 2012). From another way around, Pijanowski and Brady (2009) indicated that the salary and job benefits obtained from principalship could not compensate for their workload stress in some countries, which also is related to principals’ dissatisfaction with their profession and workplace environment (Organisation for Economic Co-operation and Development, 2020). These findings suggest a systematic investigation of the effect of working conditions on principals’ professional satisfaction.

Principals is reported to have heavy workloads in many school systems, including various instructional, administrative, and communicative tasks (Organisation for Economic Co-operation and Development, 2020). Additionally, systematic educational reforms in many countries have continuously transformed school principals’ roles and responsibilities, which has contributed to their experience of workload stress (Dicke et al., 2018b). In the TALIS 2018 framework, school principals’ workload stress was conceptualized as an imbalance between work demands and environmental or personal resources at work (Organisation for Economic Co-operation and Development, 2020); workload stress appears when the work demands placed on principals go beyond their knowledge, skills, or supports at work (Kyriacou, 2001). The indicators used in TALIS were restricted to job-related stress but did not include general anxiety or life-event stress (Viac and Fraser, 2019). TALIS 2018 results indicated that 69% of principals in OECD countries reported having too much administrative work, which was the main source of stress among principals. Particularly, in Belgium (among its French community, specifically), the Czech Republic, and Portugal, more than 90% of principals experienced this difficulty. Generally, the sources of stress reported by school leaders were fairly consistent with those reported by teachers in most countries participating in TALIS 2018 (Organisation for Economic Co-operation and Development, 2020).

A recent study (Wang et al., 2018) observed an increasing intensity and complexity of school principalship (concerning workload, overtime, and unpredictable interruptions in daily routine) in many English-speaking countries, such as Canada, the United Kingdom, and the United States. Further, heavy workload stress affects principals’ and potential principals’ physical and mental health, as well as their self-efficacy and sense of personal accomplishment, leading to negative attitudes toward the profession (Federici and Skaalvik, 2012; Bauer and Brazer, 2013; Drummond and Halsey, 2013). One study in the United States found that school principals spent 59 h per week on their school work, which was much higher than a typical workweek of 40 h (Yan, 2020). Another study in southern England reported that 43% of school principals perceived their job as either ‘very stressful’ or ‘extremely stressful’ (Phillips et al., 2007).

Apart from the effort-reward bargain issues, it has been reported that the workplace environment of school principals and teachers (particularly the academic and social climate), plays a crucial role in shaping their attitudes toward the profession, which may be placed on the spectrum from deep attachment to deep resentment of their workplace (Yan, 2020). A recent study in China detected a negative relationship between principals’ workplace attachment and their turnover intention (Shen et al., 2021). In other words, principals’ workplace attachment mitigate work stress while boosting job satisfaction (indicating a global satisfaction level with respect to their job) and consequently dampen their turnover intention (Shen et al., 2021). The TALIS 2018 measured principals’ workplace environment satisfaction by an overall evaluation of their own schools, which was high and stable in most countries, with no significant changes when compared with the 2013 survey (Organisation for Economic Co-operation and Development, 2020). Specifically, most principals “strongly agree” or “agree” with the four measures in the TALIS 2018 survey: “I am satisfied with my performance in this school” (94%); “I enjoy working at this school” (96%); “I would recommend this school as a good place to work” (95%); and “all in all, I am satisfied with my job” (95%). In some economies, such as China (specifically Shanghai), Japan, South Africa, and Turkey, principals’ workplace environment satisfaction was relatively low, with between 70 and 90% school principals rating positively on each measure. However, an earlier study in South Africa indicated that more than two-thirds of principals in disadvantaged secondary schools were dissatisfied with their workplace environment, particularly due to poor facilities and maintenance (Maforah and Schulze, 2012).

### Research Gaps in Previous Studies and Research Purposes in This Study

In human resources management, researchers and policymakers are concerned about the impacts of various working conditions, particularly professional rewards, job-related workload, and workplace environment, on employees’ professional satisfaction, but cross country differences in the effects of these factors remain largely understudied. In the last two decades, many researchers and policymakers viewed the issue of professional satisfaction from a perspective of the effort-reward bargain model (Judge et al., 2017). This is the case typically in countries governed by market mechanism, which emphasize the fundamental function of labor price in the free flow of labor force (Piracha et al., 2022). Meanwhile, more and more evidence pointed to the importance of workplace environment, particularly the soft environment such as organizational culture and climate (Yan, 2020).

Moreover, factors affecting employees’ professional satisfaction is also contingent to their position in the social ladder, or a social class rank based on income, educational attainment, occupation status, health, power, and social cognition (Kraus et al., 2013). The soft working conditions such as workplace environment is assumed to be more important to employees in relatively high positions of the social ladder, such as school principals, pursuing high-level needs such as amiability, autonomy, and accountability, while the hard conditions such as workload and job-related rewards are particularly important to employees in the low positions of the social ladder, such as nurses and cashiers, relying much on the rewards to cover their basic needs such as food and accommodation. And workers’ satisfaction with job rewards, professional workload, and workplace environment is in a continuum that if the needs in high priority are met, they will focus on those in low priority.

In current studies on principal job satisfaction, there remain two research gaps. First, there is a lack of data reflecting the multidimensional structure of principals’ job satisfaction, which limited the systematic analyses of various working conditions on principals’ professional satisfaction. Second, there is a lack of data reflecting cross country differences in principals’ job satisfaction. Partly due to these reasons, OECD included principals’ professional satisfaction, together with their workplace environment satisfaction, rewards satisfaction, and workload stress, in the TALIS 2018 survey.

In this study, we aim to explore the extent to which principals’ professional satisfaction being influenced by diverse working conditions in the 47 countries included in the TALIS 2018. We conducted a cross-country comparison based on multi-level mixed modeling that accounted for the nested structure of principals in countries. Specifically, we uncovered the general models over countries and the exact country-specific effects of workplace environment satisfaction, rewards satisfaction, and workload stress on principals’ professional satisfaction. Our findings could inform educational policy on principal retention and stimulation in each participating country and the world.

## Materials and Methods

### Survey

The TALIS is a quinquennial international survey that helps countries review and develop policies that foster the conditions necessary for effective schooling (Organisation for Economic Co-operation and Development, 2016). Given the importance of principals’ leadership in teaching and learning in schools, the TALIS facilitates the examination of principals’ roles and the support they offer to teachers and students. In TALIS 2018, 47 countries and economies participated and published their junior school data (Organisation for Economic Co-operation and Development, 2019a). In total, 9,247 principals, representing more than 300,000 school principals, took part in the aforementioned survey.

The opportunity to observe principals in 47 countries and economies allows us to investigate the relationship between principals’ satisfaction with the profession and workplace environment, rewards, and workload stress across various educational environments in a global context. The TALIS collected principals’ background, evaluative and attitudinal data (Organisation for Economic Co-operation and Development, 2019a). The data collection instruments were developed based on sources from diverse test development centers to achieve conceptually rigorous materials that had the highest possible levels of cross-cultural and cross-national diversity. Meanwhile, to ensure data quality, data collection procedures and test instruments were applied consistently throughout all regions.

In our study, the sample comprised all principals from the TALIS 2018, who provided valid information regarding their satisfaction with the profession, workplace environment, rewards, and workload stress. Since each principal from different sampling strata might represent different numbers of principals, the sampling weights given in the TALIS principal database were used to impute missing data, to calculate the country average value of each variable, to analyze the correlations, and to facilitate multilevel linear analyses.

### Measures

The present study included four job satisfaction indices, which were developed through confirmatory factor analyses and invariance testing (Organisation for Economic Co-operation and Development, 2019a). All items in these scales were measured on a four-point scale with high scores indicating high satisfaction or stress.

#### Satisfaction With Profession (PJSPRO)

The satisfaction with profession index was calculated by averaging principals’ levels of agreement (strongly disagree, disagree, agree, and strongly agree) with the following four statements: “The advantages of this profession clearly outweigh the disadvantages,” “If I could decide again, I would still choose this job/position,” “I regret that I decided to become a principal,” and “I wonder whether it would be better to choose another profession.” Confirmatory factor analysis was used in the construction of this index (*GFI* = 0.94; *SRMR* = 0.1; *RMSEA* = 0.17; *CFI* = 0.87). The overall reliability for this scale (Cronbach’s raw α) was 0.73, ranging from 0.54 (Georgia) to 0.86 (Korea and Italy), with the only outlier being Lithuania (0.25). The intraclass correlation coefficient (ICC) of the between-country variance compared to the total (both the within- and between-country variance) was 12.61%, indicating that a majority of the variance in principals’ professional satisfaction occurred at the individual level within countries, with the country level variance being small.

#### Satisfaction With Workplace Environment (PJSENV)

The satisfaction with workplace environment index was calculated by averaging principals’ levels of agreement (strongly disagree, disagree, agree, and strongly agree) with the following four statements: “I enjoy working at this school,” “I would recommend this school as a good place to work,” “I am satisfied with performance in this school,” and “All in all, I am satisfied with my job.” Confirmatory factor analysis was used in the construction of this index (*GFI* = 0.89; *SRMR* = 0.1; *RMSEA* = 0.24; *CFI* = 0.81). The overall reliability for this scale (Cronbach’s raw α) was 0.77, ranging from 0.58 (New Zealand) to 0.84 (Korea). Most of the variance in principals’ workplace environment satisfaction occurred at the individual level within countries, with the country level variance being very small (*ICC* = 7.59%).

#### Satisfaction With Rewards (REWARDS)

The satisfaction with rewards index was calculated by averaging principals’ levels of agreement (strongly disagree, disagree, agree, and strongly agree) with the following two statements: “I am satisfied with the salary I receive from my work,” and “Apart from my salary, I am satisfied with the terms of my principal contract/employment.” Confirmatory factor analysis was used in the construction of this index (*GFI* = 1; *SRMR* = 0; *RMSEA* = *N*/*A*; *CFI* = 1). The overall reliability for this scale (Cronbach’s raw α) was 0.69, ranging from 0.45 (Norway) to 0.88 (Mexico) with the only outlier being Latvia (0.13). Most of the variance in principals’ rewards satisfaction occurred at the individual level within countries (*ICC* = 13.89%).

#### Workload Stress (WORKLOAD)

The workload stress index was calculated by averaging principals’ evaluation (not at all, to some extent, quite a bit, and a lot) with the following three statements: “having too much teacher appraisal and feedback work to do,” “having too much administrative work to do,” and “having extra duties due to absent school staff.” Confirmatory factor analysis was used in the construction of this index (*GFI* = 0.98; *SRMR* = 0.06; *RMSEA* = 0.16; *CFI* = 0.93). The overall reliability for this school principal scale (Cronbach’s raw α) was 0.63, with a range between 0.32 (Sweden) and 0.77 (Colombia) in each participating country. Most of the variance in principals’ workload stress occurred at the individual level within countries (*ICC* = 15.56%).

Tables 1, 2 show the TALIS average scores and country average scores of principals’ job satisfaction with their profession, workplace environment, rewards, and workload stress, as well as the relations between these elements. For further details in each participating country, we refer to Supplementary Appendix A1.

**TABLE 1:** The TALIS average scores of principals’ job satisfaction with profession, workplace environment, rewards, and workload stress and their relations.

Individual level	*N*	RM	WM	1	2	3	4
1. Professional satisfaction	8,708	8.39 (1.51)	8.43 (8.87)	1			
2. Workplace environment satisfaction	8,716	9.22 (1.26)	9.25 (7.47)	0.56*	1		
3. Rewards	8,738	3.72 (1.03)	3.8 (6.13)	0.43*	0.31*	1	
4. Workload stress	8,746	4.38 (1.26)	4.45 (7.62)	−0.24*	−0.17*	−0.32*	1

N, number of observations; RM, raw mean before imputation; WM, weighted mean after imputation. Standard deviations are in parentheses. The weighted standard deviations are calculated as s⁢dw=∑i=1NWi⁢(Xi-X¯W)2(N`′-1)⁢∑i=1NWiN`′, where W_i is the weight for the ith observation, N′ is the number of non-zero weights, and $\bar{X_{W}}$ is the weighted mean of the observations. Imputed data with principal weights were used in the calculation of the correlation coefficients; *p < 0.0001.

**TABLE 2:** The country average scores of principals’ job satisfaction with profession, workplace environment, rewards, and workload stress and their relations.

Country level	*N*	Mean	1	2	3	4
1. Professional satisfaction	47	8.34 (0.58)	1			
2. Workplace environment satisfaction	47	9.19 (0.37)	0.72*	1		
3. Rewards	47	3.73 (0.39)	0.53*	0.44*	1	
4. Workload stress	47	4.41 (0.49)	−0.27	−0.06	−0.55*	1

N, number of participant countries; Mean, senate mean of the country average scores. Standard deviations are in parentheses. In the calculation of the country average scores, imputed data with principal weights were accounted. In the calculation of senate means and relevant standard deviations of the country average scores, each country was accounted as a senate unit, without principal weights being accounted. Standard deviations are in parentheses. Similarly, in the calculation of the correlation coefficients, each country was accounted as a senate unit, without principal weights being accounted; p* < 0.0001.

### Analyses

The proportion of missing data for each index was quite small (around 5%). No systematic missing data were found. To account for missing data, the authors used the Markov Chain Monte Carlo (MCMC) method with a single chain to create one imputation for each missing datum (Enders and Gottschall, 2011). However, the limitations of the indices in TALIS should be taken into account. For instance, a single workplace environment satisfaction measure could exhibit a bias when analyzing certain countries, which therefore should be cautiously used to explain the between-country differences in professional satisfaction.

Considering the nested structure of principals in countries in TALIS 2018 international database, we estimated the average effects of each working condition predictor on principals’ professional satisfaction over the 47 countries, together with the respective country-specific effects, via two-level linear models. In view of different analytical purposes, different selections of the predictors in these education production functions were made. The education production function used in this study can be estimated as:


$$P\,J\,S\,P\,R\,O_{i\,j}=\beta_{0}+\beta_{1}\,P\,J\,S\,E\,N\,V_{i\,j}+\beta_{2}\,R\,E\,W\,A\,R\,D\,S_{i\,j}+\beta_{3}\,W\,O\,R\,K\,L\,O\,A\,D_{i\,j}+\mu_{j}+\varepsilon_{i\,j}$$

Where *PJSPRO*_*ij*_ is the professional satisfaction score of the principal *i* in country *j*, *PJSENV*_*ij*_ is the measure of principal’s workplace environment satisfaction, *REWARDS*_*ij*_ is the measure of principal’s job rewards satisfaction, *WORKLOAD*_*ij*_ is the measure of principal’ workload stress, μ_*j*_ and ε_*ij*_ are the error terms at the country and individual level in relation to the principal’s professional satisfaction.

SAS 9.4 software was used to impute missing data and to conduct descriptive and multilevel linear analyses.

## Results

### Fixed and Country-Specific Effects of Workplace Environment Satisfaction

From Model 1 in Table 3, we can see that the fixed effect of workplace environment satisfaction is 0.61 (95% CI [0.57, 0.65]), with all country-specific effects being positive. Specifically, most country-specific effects are not significantly different from the fixed effects, except those in Chile (0.44; 95% CI [0.28, 0.6]), Viet Nam (0.51; 95% CI [0.37, 0.65]), and Mexico (0.52; 95% CI [0.4, 0.64]), which are significantly smaller, and those in Russian Federation (0.71; 95% CI [0.59, 0.83]) and England (0.75; 95% CI [0.57, 0.93]), which are significantly larger.

**TABLE 3:** Predicting principals’ professional satisfaction by their workplace environment satisfaction, rewards satisfaction, and workload stress.

Parameter	Model 0	Model 1	Model 2	Model 3
Intercept	8.34* (0.09)	2.72* (0.22)	6.35* (0.16)	9.61* (0.08)
Workplace environment		0.61* (0.02)		
Rewards			0.53* (0.03)	
Workload stress				−0.29* (0.02)
Variance components				
Between-country	0.3	0.94	0.81	0.09
Within-country	68.7	48.49	58.49	64.47
Variance explained (%)				
Between-country^Δ^		−213.33%	−170%	70%
Within-country^Δ^		29.42%	14.86%	6.16%
Fit statistics				
−2 Res Log Likelihood	41,906	38,743	40,395	41,216
AIC	41,910	38,751	40,403	41,224

Standard errors are in parentheses. ^Δ^Compared to Model 0; p* < 0.001.

**FIGURE 1:**
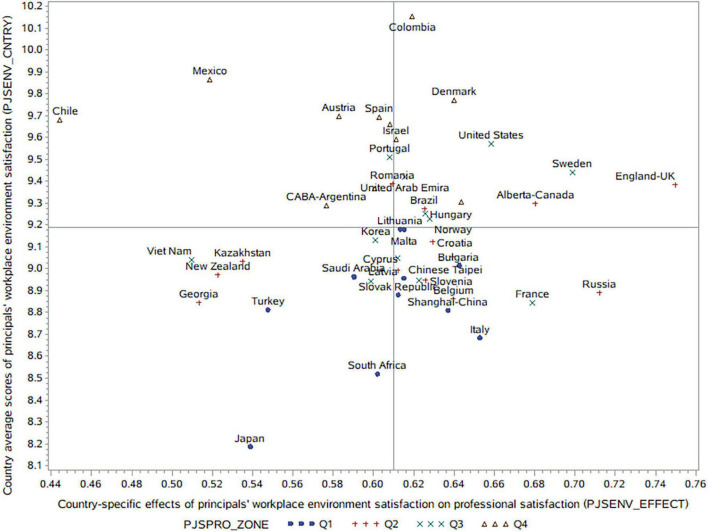
Principals’ workplace environment satisfaction and its effects on their professional satisfaction in each country.

Figure 1 shows the distribution of the country average scores of principals’ workplace environment satisfaction (PJSENV_CNTRY) and the country-specific effects of principals’ workplace environment satisfaction on professional satisfaction (PJSENV_EFFECT), with the quartile ranges of the country average scores of principals’ professional satisfaction (PJSPRO_ZONE, Q1 is the lowest quartile and Q4 is the highest quartile) being labeled simultaneously. From this Figure we can see the consistency between principals’ satisfaction with the profession and workplace environment at country level (*r* = 0.72; *p* = 0.001). For instance, countries with high workplace environment satisfaction among principals are more likely to have principals with high professional satisfaction (Q4), as is the case for Colombia, Mexico, Chile, Denmark, Austria, Spain, Singapore, and Israel. Similarly, countries with low workplace environment satisfaction among principals tend to have principals with low professional satisfaction (Q1), as is the case for Japan, South Africa, Italy, Turkey, and Shanghai-China. These findings indicate the potential of workplace environment improvement on principals’ professional satisfaction at country level.

Table 3 also shows that, in Model 1, workplace environment satisfaction explains 29.42% of the within-country variance in principals’ professional satisfaction, with the between-country variance being inflated dramatically (−213.33%, compared with Model 0). This inconsistency between individual and country level variance explained by principals’ workplace environment satisfaction indicates that although principals’ individual professional satisfaction might benefit from improvements to their workplace environment, principals in countries with higher professional satisfaction do not necessarily benefit more from workplace environment improvements at the system level (*r* = −0.14; *p* = 0.351). That is, the country-specific effects of workplace environment on professional satisfaction are not significantly higher in countries with high professional satisfaction among principals.

Moreover, in most countries with extreme values in professional satisfaction (Q1 and Q4) and workplace environment satisfaction among principals, the country-specific effects of workplace environment on professional satisfaction tend to be at relatively low or medium levels. Further, in the countries with medium values in these two scales, the country-specific effects spread even more widely. Specifically, principals’ professional satisfaction is more sensitive to workplace environment changes in the following regions: England-United Kingdom, Russian Federation, Sweden, Alberta-Canada, France, and the United States, most of which are developed countries. While principals’ professional satisfaction in Chile, Mexico, Viet Nam, Kazakhstan, New Zealand, Georgia, Turkey, and Japan is less sensitive to changes in workplace environment.

### Fixed and Country-Specific Effects of Rewards Satisfaction

Table 3 also shows that, in Model 2, the fixed effect of rewards satisfaction is 0.53 (95% CI [0.47, 0.59]), with all country-specific effects being positive. Specifically, most country-specific effects are not significantly different from the fixed effect, with those in Israel (0.31; 95% CI [0.03, 0.57]), Colombia (0.3; 95% CI [0.1, 0.68]), Spain (0.37; 95% CI [0.11, 0.59]), Chile (0.37; 95% CI [0.15, 0.59]), and Mexico (0.39; 95% CI [0.23, 0.63]) being significantly smaller and those in Russian Federation (0.85; 95% CI [0.67, 1.03]) and Saudi Arabia (0.74; 95% CI [0.54, 0.94]) being significantly larger.

**FIGURE 2:**
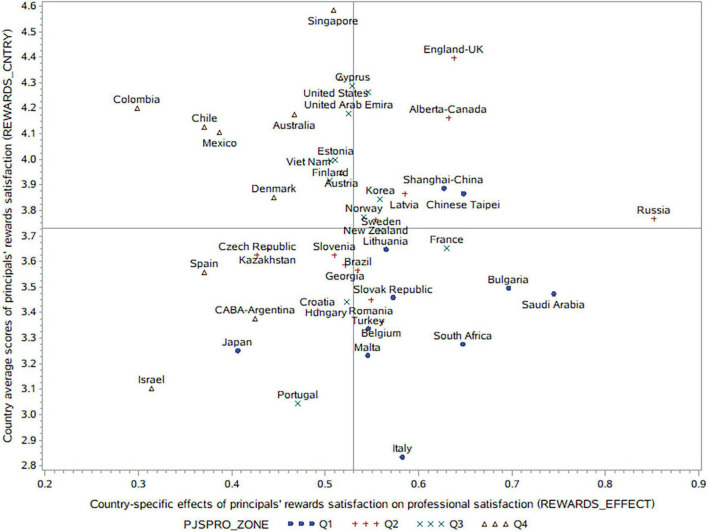
Principals’ rewards satisfaction and its effects on their professional satisfaction in each country.

Figure 2 shows the distribution of the country average scores of rewards satisfaction among principals (REWARDS_CNTRY), as well as the country-specific effects of principals’ rewards satisfaction on professional satisfaction (REWARDS_EFFECT), with the quartile ranges of the country average scores of principals’ professional satisfaction (PJSPRO_ZONE) being labeled simultaneously. Generally, a country level consistency between rewards and professional satisfaction among school principals appears in many countries (*r* = 0.53; *p* = 0.001). For instance, some countries have high rewards and professional satisfaction simultaneously (such as Singapore, Netherlands, Colombia, Australia, Chile, Mexico, and Austria). Further, some countries have low rewards and professional satisfaction simultaneously (such as Italy, Malta, Japan, South Africa, and Turkey). Conversely, some countries have relatively high rewards satisfaction but low professional satisfaction, such as England-United Kingdom and Alberta-Canada while some countries have relatively low rewards satisfaction but high professional satisfaction, such as Portugal and Israel. These findings indicate the partial effect of job rewards on professional satisfaction at country level. Other factors, such as workplace environment changes, should be accompanied with systematic improvement of principals’ professional satisfaction.

From Model 2 in Table 3, we can see that rewards satisfaction explains 14.86% of the within-country variance in principals’ professional satisfaction, with the between-country variance being inflated dramatically (−170%, compared with Model 0). That is, accounting for the country-specific effects of rewards satisfaction on professional satisfaction, principals’ individual professional satisfaction might benefit from improving job rewards, while principals in countries with higher professional satisfaction tend to benefit less from systematic improvements to job rewards (*r* = −0.64; *p* = 0.001). Specifically, countries with high professional satisfaction (Q4), such as Colombia, Israel, Chile, Mexico, Spain, CABA-Argentina, Denmark, and Australia, exhibit a smaller effect of rewards on professional satisfaction. Further, countries with low professional satisfaction exhibit a larger effect of rewards on professional satisfaction, including several Q1 regions, such as Saudi Arabia, Bulgaria, Chinese Taipei and Shanghai-China, as well as several Q2 regions, such as Russian Federation, England-United Kingdom, and Alberta-Canada.

### Fixed and Country-Specific Effects of Workload Stress

From Model 3 in Table 3, we can see that the fixed effect of workload stress is −0.29 (95% CI [−0.33, −0.25]), with all country-specific effects being negative. Specifically, most country-specific effects are not significantly different from the fixed effect, with those in Saudi Arabia (−0.45; 95% CI [−0.52, −0.36]), Bulgaria (−0.44; 95% CI [−0.56, −0.32]), Turkey (−0.38; 95% CI [−0.48, −0.28]), Japan (−0.36; 95% CI [−0.48, −0.24]), and Russian Federation (−0.36; 95% CI [−0.46, −0.26]) being significantly lower, that is, large negative effects and those in Mexico (−0.21; 95% CI [−0.31, −0.11]), United States (−0.2; 95% CI [−0.3, −0.1]), Chile (−0.19; 95% CI [−0.31, −0.07]), and Colombia (−0.13; 95% CI [−0.23, −0.03]) being significantly higher, that is, small negative effects. Specifically, many of the countries with small negative effects are located on the American continent.

**FIGURE 3:**
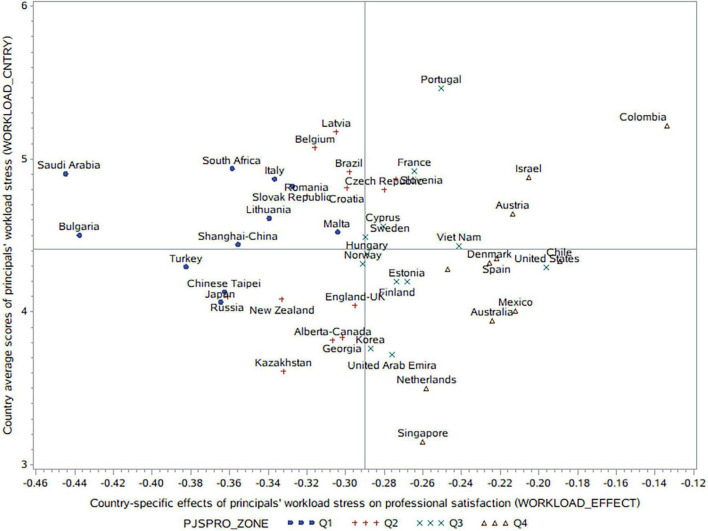
Principals’ workload stress and its effects on their professional satisfaction in each country.

Figure 3 indicates an inconsistency between principals’ workload stress and professional satisfaction at country level (*r* = −0.27; *p* = 0.16). For instance, some countries show high workload stress and professional satisfaction scores simultaneously (e.g., Colombia, Israel, and Australia), while other countries show low workload stress and high professional satisfaction scores (e.g., Singapore and Netherlands). The success of these countries is worth further exploration. In contrast, some regions have low workload stress and professional satisfaction simultaneously (such as Russian Federation, Japan, and Chinese Taipei) while some countries have high workload stress but low professional satisfaction scores (such as South Africa, Saudi Arabia, Italy, and Slovak Republic).

From Model 3 in Table 3, we can see that compared with the unconditional model (Model 0), workload stress explains 70% of the between-country variance and 6.16% of the within-country variance in professional satisfaction. That is, workload stress is much a system level predictor for principals’ professional satisfaction. Principals in countries with higher professional satisfaction scores tend to be less affected by heavy workload stress.

In Figure 3 we can see the high consistency between the country average scores of professional satisfaction and the country-specific effects of workload stress on professional satisfaction (*r* = 0.92; *p* = 0.001). Specifically, in countries with small negative effects of workload stress on professional satisfaction, the country average scores of professional satisfaction are among the highest (Q4), as shown in the right part of Figure 3. These regions include Colombia, Chile, Israel, Mexico, Australia, Denmark, Spain, Austria, CABA-Argentina, Singapore, and Netherlands. In countries with large negative effects of workload stress on professional satisfaction, the country average scores of principal professional satisfaction are among the lowest (Q1), as shown in the left part of Figure 3. These regions include Saudi Arabia, Bulgaria, South Africa, Japan, Turkey, Chinese Taipei, Italy, Shanghai-China, Lithuania, Slovak Republic, and Malta.

## Conclusion and Discussion

Being a school principal is considered a way of self-actualization, with its meaning and function as the core reasons behind job satisfaction (Grissom and Brendan, 2018). The results from the TALIS 2018 indicated that although principals in many regions tend to have high professional satisfaction in general, especially in tandem with high workplace environment and/or rewards satisfaction, they also tend to have high workload stress (Organisation for Economic Co-operation and Development, 2020). However, the challenging workplace environment, insufficient rewards, and heavy workload in some countries make principalship a difficult profession. Despite the extensive interest of educational researchers and policymakers in improving principals’ satisfaction with the profession and relevant working conditions, little is known about the general effects of principals’ working condition satisfactions on their professional satisfaction over countries and the efficient methods in specific countries. The current study aims to fill these research gaps in literature via multi-level linear analyses based on the TALIS 2018 data.

### Dimensions of Working Conditions Differ in Their Potentials to Impact Principals’ Professional Satisfaction

This study confirmed our basic hypothesis that compared to job rewards and workload stress, workplace environment is more important to school principals, who are in relatively high position on social ladder. About one third of the individual level differences in principals’ professional satisfaction (29.42%) is related to their satisfaction with workplace environment, with 14.86% being related to their satisfaction with professional rewards and only 6.16% being related to their workload stress. Meanwhile, principals’ workplace environment satisfaction could be a strong predictor for their professional satisfaction in countries with different levels of professional satisfaction among principals. This is especially important for some regions with high professional satisfaction among principals (Q4), such as Australia, Colombia, Denmark, and Israel, wherein rewards satisfaction and workload stress tends to impact principals’ professional satisfaction to a lesser extent. This is also true for some Q2 and Q3 regions, such as the Czech Republic, Slovenia, Cyprus, Finland, Hungary, and United Arab Emirates.

More specifically, principals’ professional experience is largely related to their workplace environment. An improvement in workplace environment is much efficient to principals’ professional satisfaction. In contrast, principals’ professional experience is weakly related to their workload stress. This finding is quite informative to policymakers in some Asian countries, such as China and Korea, which have school balance policies via transferring excellent principals to weak schools for improvement reason (Shen et al., 2021; Wang et al., 2021). The educational authorities can spend more resources on workplace environment improvement while ignore to some extent the heavy workload in school improvement. This combined policy is much likely to keep principals’ professional satisfaction.

Apart from workplace environment satisfaction, the general levels of principals’ professional satisfaction in each country could be considered as a background to select efficient methods to enhance principals’ professional satisfaction. In line with multiple previous studies (e.g., Tran, 2017; Yan, 2020), our study found that principals are more likely to be satisfied with their profession if they are offered with better job rewards in all TALIS 2018 participating regions. One unique finding from this cross-country comparative study is that this market mechanism, emphasizing the fundamental function of labor price in the free flow of labor force, is especially important in regions with low professional satisfaction levels among principals (Q1 and Q2). Further, providing principals with better job rewards appears to be an effective method to improve their professional satisfaction at country level, considering the fact that principals’ rewards satisfaction is also positively related to their professional satisfaction at country level.

Future studies should give more attention to regions where there is an inconsistency between job rewards and professional satisfaction at system level. In regions with high rewards but low professional satisfaction among principals, such as England-United Kingdom and Alberta-Canada, the country-specific effects of rewards on professional satisfaction tend to be high, which might be one of the policy considerations for high rewards at country level. Additional tasks, such as workplace environment improvement and workload reduction, should be considered to improve principals’ professional satisfaction systematically. Similarly, in regions with low rewards but high professional satisfaction among principals, such as Portugal, Israel, CABA-Argentina, and Spain, the country-specific effects of rewards on professional satisfaction are low, which might drive policymakers to devise other methods (apart from rewards) for improving principals’ professional satisfaction. These findings highlight the importance of developing systematic approaches to examine the potential for policy implications of working conditions, since the overall improvement of professional satisfaction at country level cannot be confirmed at the individual level. Specifically, conclusions derived from individual level analyses do not necessarily apply at country level, and may even be contradictory.

Comparatively, workload stress is more of a system level predictor for between-country differences in professional satisfaction, with 70% of the between-country variance being explained. The country level differences in principals’ professional satisfaction are largely related to the differential effect sizes of workload stress on professional satisfaction. Similar with the differential effect sizes of job rewards satisfaction on professional satisfaction, in countries with high levels of professional satisfaction (Q3 and Q4), the negative effect sizes of workload stress on professional satisfaction tend to be small, as is the case for some American countries (Mexico, United States, Chile, and Colombia). While in regions with low professional satisfaction among principals (Q1 and Q2), the sensitivity of professional satisfaction to workload stress tends to be high. Thereafter, heavy workload stress may indeed be the “straw that breaks the camel’s back.” Moreover, high workload stress leads to an increasing reluctance among experienced teachers to assume leadership roles (as is the case for Germany and the United States) and a trend of aging principals (as is the case for Korea, Italy, and New Zealand) (Snodgrass Rangel, 2018; Organisation for Economic Co-operation and Development, 2020; Pietsch et al., 2020; Yan, 2020). In contrast, the current study indicated an inconsistency between professional satisfaction and workload stress at country level, as Q3 and Q4 countries do not necessarily have low levels of workload stress, suggesting the comprehensive effects of workload stress and other factors on principals’ professional satisfaction at country level.

### Multiple Dimensions of Working Conditions Work Together in Shaping Principals’ Professional Satisfaction

From the current study we can see evidence that workplace environment, job rewards, and workload stress could be strong predictors for principals’ professional satisfaction in specific countries simultaneously. The evidence is inconsistent with the traditional theories on the job satisfaction-dissatisfaction continuum that certain working conditions lead to employees’ satisfaction when they are present and dissatisfaction when they are absent (Newby, 1999). Table 4 summarizes the overlap of the high halves of the country-specific effects of workplace environment satisfaction, rewards satisfaction, and workload stress, with the quartile ranges of the country average scores of principals’ professional satisfaction (PJSPRO_ZONE) being labeled simultaneously. For example, the country-specific effect of rewards on professional satisfaction is higher than average in Korea, while those of workplace environment satisfaction and workload stress are lower than average. This finding indicates that providing financial and contractual incentives could be an efficient way to attract principals to work in specific schools in Korea.

**TABLE 4:** The higher than the averages country-specific effects of workplace environment satisfaction, rewards satisfaction, and workload stress in TALIS 2018.

Country	High workplace environment satisfaction effect	High rewards satisfaction effect	High workload stress effect	Professional satisfaction level
Bulgaria	Yes	Yes	Yes	Q1
Chinese Taipei	Yes	Yes	Yes	Q1
Italy	Yes	Yes	Yes	Q1
Lithuania	Yes	Yes	Yes	Q1
Malta	Yes	Yes	Yes	Q1
Shanghai-China	Yes	Yes	Yes	Q1
Slovak Republic	Yes	Yes	Yes	Q1
Alberta-Canada	Yes	Yes	Yes	Q2
Belgium	Yes	Yes	Yes	Q2
Brazil	Yes	Yes	Yes	Q2
England-United Kingdom	Yes	Yes	Yes	Q2
Latvia	Yes	Yes	Yes	Q2
Russian Federation	Yes	Yes	Yes	Q2
Norway	Yes	Yes	Yes	Q3
France	Yes	Yes		Q3
Sweden	Yes	Yes		Q3
United States	Yes	Yes		Q3
Croatia	Yes		Yes	Q2
Czech Republic	Yes			Q2
Slovenia	Yes			Q2
Cyprus	Yes			Q3
Finland	Yes			Q3
Hungary	Yes			Q3
United Arab Emirates	Yes			Q3
Australia	Yes			Q4
Colombia	Yes			Q4
Denmark	Yes			Q4
Israel	Yes			Q4
Saudi Arabia		Yes	Yes	Q1
South Africa		Yes	Yes	Q1
Turkey		Yes	Yes	Q1
New Zealand		Yes	Yes	Q2
Romania		Yes	Yes	Q2
Korea		Yes		Q3
Japan			Yes	Q1
Georgia			Yes	Q2
Kazakhstan			Yes	Q2
Estonia				Q3
Portugal				Q3
Viet Nam				Q3
Austria				Q4
CABA-Argentina				Q4
Chile				Q4
Mexico				Q4
Netherlands				Q4
Singapore				Q4
Spain				Q4

In line with the findings in previous studies from some countries (e.g., Theorell, 2017), the current study indicated that many of the high country-specific effects of workplace environment satisfaction, rewards satisfaction, and workload stress overlap. For instance, in France, Sweden, and the United States, the country-specific effects of workplace environment and rewards are high. Additionally, in New Zealand, Romania, Saudi Arabia, South Africa, and Turkey, the country-specific effects of workplace environment satisfaction and workload stress are high. Further, in Croatia, the country-specific effects of workplace environment and workload stress are high.

Several regions exhibit higher country-specific effects for workplace environment satisfaction, rewards satisfaction, and workload stress than their respective TALIS average size, indicating a large potential to improve principals’ professional satisfaction. These regions include Alberta-Canada, Belgium, Brazil, Bulgaria, Chinese Taipei, England-United Kingdom, Italy, Lithuania, Latvia, Malta, Norway, Russian Federation, Shanghai-China, and Slovak Republic. These countries (except Norway) exhibit relatively low levels of professional satisfaction among principals (Q1 and Q2). Additionally, several regions exhibit lower country-specific effects for workplace environment satisfaction, rewards satisfaction, and workload stress than their respective average size; therefore, further exploration is required to improve principals’ professional satisfaction. These regions include Austria, CABA-Argentina, Chile, Estonia, Mexico, Netherlands, Portugal, Singapore, Spain, and Viet Nam. These regions exhibit relatively high levels of professional satisfaction among principals (Q3 and Q4).

### Strengths, Limitations, and Future Prospects

Countries and economies might cherish different methods to stimulate and retain school principals’ professional sprit and satisfaction. The TALIS 2018 is a valuable tool to assess the overall relationship between professional satisfaction and working conditions among school principals. One valuable contribution of this study is that it highlights the influence of working conditions on principals’ professional satisfaction from a cross-country perspective that allows us to identify the general trends in this topic. Another contribution of this study is that it provides clues for a systematic improvement of principals’ professional satisfaction at country level via identifying country level predictors. Specifically, improving principals’ workplace environment and rewards is likely to improve their professional satisfaction at both the individual and system levels. Further, principals’ workload stress is negatively related to their professional satisfaction only at the individual level, with the country-specific effects being high in countries with low levels of professional satisfaction. These findings seem to be valid and have cross-cultural sustainability. No clear social or cultural clue concerning the relationship between working conditions and professional satisfaction among principals at both the individual and country levels was found in this study.

Though our study takes advantage of large-scale international data and rigorous data analyses, it still has limitations and suggests future prospects. First, we only worked with data of principals who were working at the time of the survey. Therefore, we could not compare principals’ professional satisfaction before, during and after their tenure. Similar challenges have been reported in previous research, specifically concerning principals’ reasoning for leaving their jobs (e.g., Tran, 2017; Yan, 2020). Further, the self-reported data of school principals in one measurement point might carry some bias across countries due to social and cultural differences in certain conceptual constructs. For example, principals’ understanding about the importance of each indicator of professional satisfaction differs across countries.

Second, this study adopted multilevel linear models to investigate how each working condition predictor influence principals’ professional satisfaction via TALIS 2018 data. However, the three facets of working condition satisfaction might interact with one another in some countries, such as in the United States (Yan, 2020). Thus, future studies should explore the interactive and moderating effects of working conditions on principals’ professional satisfaction in specific countries as they are influenced highly by two or three predictors simultaneously. Specifically, these future studies could prove helpful to explore the sensitivity of workload stress on professional satisfaction in countries like Saudi Arabia, Bulgaria, and Turkey.

Third, this study did not consider all sources of principal profession satisfaction, such as social prestige and relationships, training and professional development, school autonomy, and institutional supports (Organisation for Economic Co-operation and Development, 2020). Neither did it control principals’ background and their work contextual variables such as the economic, social and cultural conditions in participating countries and the world, which related to principals’ job satisfaction. Previous studies indicated that social prestige is an important factor in the allure of teaching careers among trainee teachers and can improve the retention ratio of effective teachers (Price and Weatherby, 2018). Professions with high social prestige are more likely to attract and satisfy promising candidates, as is the case in medicine or engineering (Kraus et al., 2013). A specific context of principals’ job satisfaction worth further exploration is the COVID 19 Pandemic, which changes principals’ workplace, workload, and welfare simultaneously (Harris and Jones, 2020; Hayes et al., 2021).

Last but not least, this study emphasized the multidimensional construct of principals’ job satisfaction comprising the satisfaction with the profession and its diverse working conditions, which work together in shaping principals’ professional well-being and retention (Wang et al., 2021). However, it ignored some core characteristics of the profession, such as its variety, autonomy, feedback, significance, and identity (Hackman and Oldham, 1976). It will be much informative if future study could examine the contribution of these characteristics to principals’ professional satisfaction and also the impacts of diverse working conditions on them at individual and country level.

## Data Availability Statement

The datasets presented in this study can be found in online repositories. The names of the repository/repositories and accession number(s) can be found below: https://www.oecd.org/education/talis/talis-2018-data.htm.

## Ethics Statement

The studies involving human participants were reviewed and approved by OECD. The patients/participants provided their written informed consent to participate in this study.

## Author Contributions

BN contributed to problem statement, methods and statistical models, interpretation and discussion of results, and discussion and conclusion. HL contributed to conceptual framework and style and structure review. YC contributed to data analysis. All authors contributed to the article and approved the submitted version.

## Conflict of Interest

The authors declare that the research was conducted in the absence of any commercial or financial relationships that could be construed as a potential conflict of interest.

## Publisher’s Note

All claims expressed in this article are solely those of the authors and do not necessarily represent those of their affiliated organizations, or those of the publisher, the editors and the reviewers. Any product that may be evaluated in this article, or claim that may be made by its manufacturer, is not guaranteed or endorsed by the publisher.
