# Supplementary material for: Working Conditions Influencing Junior School Principalship as a Satisfying Profession: A Cross-Country Comparative Study
Source: Front Psychol. 2022 Mar 25;13:834349. doi: 10.3389/fpsyg.2022.834349 (PMC8990902; doi:10.3389/fpsyg.2022.834349)
Supplement: Supplementary file 1 [file Data_Sheet_1.docx]

**Appendix A1**

*Sample Size and Number of Observations, Country Average Score, and Standard Deviation of Principals’ Job Satisfaction with Profession, Workplace Environment, Rewards, and Workload Stress in Each Participating Country of TALIS 2018*

| Country | Sample size | Professional satisfaction | | | Workplace environment satisfaction | | | Rewards satisfaction | | | Workload stress | | |
| --- | --- | --- | --- | --- | --- | --- | --- | --- | --- | --- | --- | --- | --- |
|  |  | N | Mean | STD | N | Mean | STD | N | Mean | STD | N | Mean | STD |
| Alberta-Canada | 129 | 120 | 8.07 | 4.79 | 121 | 9.30 | 3.25 | 120 | 4.16 | 2.51 | 122 | 10.02 | 7.70 |
| Australia | 238 | 205 | 8.92 | 4.16 | 206 | 9.31 | 3.87 | 206 | 4.18 | 2.57 | 207 | 10.32 | 6.97 |
| Austria | 277 | 270 | 9.03 | 3.43 | 270 | 9.70 | 2.60 | 268 | 3.95 | 2.16 | 269 | 10.50 | 5.24 |
| Belgium | 308 | 282 | 7.90 | 2.90 | 282 | 8.86 | 2.23 | 285 | 3.36 | 1.81 | 282 | 12.21 | 4.32 |
| Brazil | 186 | 183 | 8.22 | 26.25 | 183 | 9.27 | 23.26 | 183 | 3.57 | 19.68 | 181 | 11.73 | 52.15 |
| Bulgaria | 200 | 192 | 7.06 | 5.39 | 195 | 9.02 | 3.46 | 194 | 3.50 | 2.38 | 193 | 11.32 | 7.95 |
| CABA-Argentina | 134 | 114 | 8.92 | 2.85 | 117 | 9.29 | 2.42 | 117 | 3.38 | 2.25 | 117 | 10.21 | 5.48 |
| Chile | 179 | 167 | 9.21 | 6.69 | 168 | 9.68 | 7.98 | 167 | 4.13 | 6.25 | 168 | 10.68 | 16.25 |
| Chinese Taipei | 202 | 201 | 7.73 | 3.28 | 201 | 8.96 | 2.84 | 201 | 3.87 | 1.95 | 197 | 10.61 | 4.40 |
| Colombia | 157 | 137 | 9.39 | 9.89 | 138 | 10.15 | 8.15 | 137 | 4.20 | 8.37 | 138 | 12.38 | 28.44 |
| Croatia | 195 | 183 | 8.13 | 2.95 | 184 | 9.12 | 2.51 | 185 | 3.41 | 2.23 | 184 | 11.69 | 4.22 |
| Cyprus | 89 | 85 | 8.53 | 1.52 | 83 | 9.05 | 1.35 | 85 | 4.29 | 0.91 | 85 | 11.17 | 2.66 |
| Czech Republic | 219 | 217 | 8.22 | 4.43 | 217 | 9.05 | 3.44 | 217 | 3.63 | 3.07 | 217 | 11.82 | 7.28 |
| Denmark | 148 | 136 | 9.09 | 4.36 | 135 | 9.77 | 3.77 | 135 | 3.85 | 3.25 | 136 | 10.54 | 7.59 |
| England-UK | 157 | 146 | 8.28 | 8.05 | 143 | 9.39 | 5.42 | 143 | 4.40 | 4.38 | 143 | 11.02 | 11.89 |
| Estonia | 195 | 192 | 8.69 | 1.74 | 192 | 8.94 | 1.71 | 192 | 4.00 | 1.15 | 191 | 10.59 | 3.71 |
| Finland | 148 | 148 | 8.57 | 3.09 | 148 | 8.95 | 2.56 | 148 | 3.92 | 1.84 | 148 | 10.33 | 4.43 |
| France | 196 | 185 | 8.42 | 9.51 | 183 | 8.85 | 7.93 | 185 | 3.65 | 6.24 | 186 | 12.18 | 15.71 |
| Georgia | 192 | 168 | 8.23 | 4.20 | 169 | 8.85 | 4.45 | 170 | 3.59 | 2.68 | 169 | 8.94 | 9.32 |
| Hungary | 189 | 180 | 8.47 | 5.32 | 180 | 9.25 | 3.91 | 180 | 3.44 | 3.67 | 179 | 10.49 | 9.53 |
| Israel | 187 | 153 | 8.90 | 3.30 | 152 | 9.59 | 2.83 | 153 | 3.10 | 2.71 | 153 | 12.00 | 6.70 |
| Italy | 192 | 186 | 7.75 | 9.61 | 186 | 8.68 | 6.98 | 187 | 2.83 | 5.20 | 186 | 12.49 | 13.02 |
| Japan | 196 | 192 | 7.86 | 10.12 | 194 | 8.19 | 9.95 | 194 | 3.25 | 6.88 | 193 | 11.11 | 16.43 |
| Kazakhstan | 331 | 331 | 8.22 | 5.26 | 331 | 9.03 | 4.97 | 331 | 3.65 | 4.02 | 331 | 9.32 | 11.34 |
| Korea | 165 | 147 | 8.43 | 7.46 | 148 | 9.13 | 7.01 | 147 | 3.85 | 5.07 | 147 | 9.67 | 11.01 |
| Latvia | 137 | 133 | 7.93 | 2.78 | 133 | 8.99 | 1.97 | 134 | 3.87 | 1.25 | 133 | 12.73 | 3.85 |
| Lithuania | 195 | 192 | 7.72 | 2.31 | 192 | 9.18 | 2.25 | 194 | 3.65 | 1.82 | 194 | 11.84 | 3.87 |
| Malta | 58 | 52 | 7.68 | 1.53 | 50 | 9.18 | 1.04 | 51 | 3.23 | 1.08 | 52 | 11.51 | 2.21 |
| Mexico | 193 | 189 | 9.31 | 11.61 | 193 | 9.86 | 11.29 | 192 | 4.11 | 11.42 | 193 | 9.41 | 25.45 |
| Netherlands | 127 | 118 | 8.96 | 2.53 | 119 | 9.37 | 2.49 | 119 | 4.32 | 1.55 | 117 | 8.71 | 4.64 |
| New Zealand | 190 | 183 | 8.15 | 4.24 | 183 | 8.97 | 3.52 | 183 | 3.76 | 3.16 | 180 | 10.37 | 8.32 |
| Norway | 193 | 156 | 8.39 | 3.78 | 157 | 9.23 | 2.57 | 158 | 3.77 | 2.19 | 158 | 10.49 | 5.77 |
| Portugal | 200 | 200 | 8.44 | 3.64 | 200 | 9.51 | 2.51 | 199 | 3.05 | 2.85 | 198 | 13.93 | 4.33 |
| Romania | 199 | 198 | 8.03 | 6.70 | 198 | 9.39 | 6.30 | 198 | 3.45 | 4.16 | 198 | 11.42 | 13.01 |
| Russia | 230 | 228 | 8.30 | 15.59 | 228 | 8.89 | 11.56 | 229 | 3.77 | 9.36 | 229 | 10.13 | 27.80 |
| Saudi Arabia | 195 | 179 | 6.95 | 10.25 | 176 | 8.96 | 8.14 | 177 | 3.47 | 6.61 | 179 | 11.71 | 17.25 |
| Shanghai-China | 198 | 197 | 7.54 | 2.34 | 197 | 8.81 | 2.19 | 197 | 3.89 | 1.64 | 196 | 11.45 | 4.14 |
| Singapore | 169 | 163 | 9.29 | 1.22 | 166 | 9.66 | 1.27 | 167 | 4.59 | 1.01 | 167 | 8.73 | 2.71 |
| Slovak Republic | 180 | 178 | 7.83 | 3.96 | 177 | 8.88 | 2.95 | 179 | 3.46 | 2.35 | 176 | 11.88 | 6.21 |
| Slovenia | 134 | 117 | 8.38 | 2.45 | 117 | 8.95 | 1.84 | 117 | 3.62 | 1.70 | 117 | 11.78 | 4.15 |
| South Africa | 177 | 164 | 7.77 | 10.74 | 163 | 8.52 | 9.94 | 165 | 3.28 | 7.61 | 164 | 12.40 | 19.08 |
| Spain | 399 | 392 | 8.91 | 5.40 | 394 | 9.70 | 4.46 | 394 | 3.56 | 3.89 | 395 | 11.17 | 9.74 |
| Sweden | 183 | 153 | 8.55 | 5.50 | 151 | 9.44 | 4.30 | 153 | 3.72 | 2.90 | 154 | 10.40 | 7.36 |
| Turkey | 198 | 189 | 7.41 | 16.09 | 189 | 8.81 | 14.66 | 191 | 3.34 | 9.52 | 190 | 10.72 | 27.02 |
| United Arab Emirates | 521 | 457 | 8.74 | 1.60 | 457 | 9.42 | 1.51 | 458 | 4.18 | 1.13 | 454 | 9.58 | 2.96 |
| United States | 166 | 155 | 8.87 | 26.52 | 154 | 9.57 | 20.08 | 157 | 4.27 | 17.22 | 156 | 10.13 | 47.63 |
| Viet Nam | 196 | 195 | 8.70 | 7.74 | 196 | 9.04 | 8.88 | 196 | 4.00 | 5.75 | 195 | 10.93 | 20.14 |

*Note.* N=number of observations in each participating country. Mean=weighted mean after imputation. STD=standard deviation. In the calculation of the country average scores, imputed data with principal weights were accounted.
